# Vascular endothelial growth factor improves the therapeutic effects of cyclodextrin in Niemann-Pick type C mice

**DOI:** 10.1080/19768354.2019.1651768

**Published:** 2019-08-25

**Authors:** Min Seock Jeong, Jae-sung Bae, Hee Kyung Jin

**Affiliations:** a KNU Alzheimer’s disease Research Institute, Kyungpook National University, Daegu, Korea; b Department of Laboratory Animal Medicine, College of Veterinary Medicine, Kyungpook National University, Daegu, Korea; c Department of Physiology, Cell and Matrix Research Institute, School of Medicine, Kyungpook National University, Daegu, South Korea; d Department of Biomedical Science, BK21 Plus KNU Biomedical Convergence Program, Kyungpook National University, Daegu, Korea

**Keywords:** Niemann-Pick type C disease mouse, vascular endothelial growth factor, neuroinflammation, 2-hydroxypropyl-β-cyclodextrin, combination therapy

## Abstract

Niemann-Pick type C disease (NP-C) is a fatal neurodegenerative disorder caused by a deficiency in the function of the *NPC1* gene. Malfunction of this gene/protein leads to progressive accumulation of unesterified cholesterol and sphingolipids in many organs, including the brain. To date, drugs that target pivotal stages in the pathogenic cascade have been tested as monotherapies or in combination with a second agent, showing additive benefits. In this study, we have investigated the effects of combining centrally and systemically administered therapies in a mouse model of NP-C, i.e. overexpression of brain-specific vascular endothelial growth factor (VEGF) in combination with systemic administration of 2-hydroxypropyl-β-cyclodextrin (CD). We found that animals treated using a combination of VEGF and CD showed an improvement in pathophysiology compared to those treated with CD alone or brain VEGF overexpression alone, or non-treated NP-C mice. Combination therapy increased the time period over which NP-C mice maintained their body-weight and motor function, and decreased the abnormal accumulation of lipids. In addition, combination therapy delayed the onset of Purkinje cell loss and reduced neuroinflammation. Taken together, our results demonstrate that combination therapy using VEGF and CD is a promising therapeutic modality for treating NP-C, and suggest that it represents a potential strategy for the treatment of diseases that cause both visceral and brain pathologies.

## Introduction

Niemann-Pick type C disease (NP-C) is a neurovisceral autosomal recessive lysosomal storage disease caused by mutations in the *NPC1* gene. Defects in NPC1 protein result in abnormal intracellular traﬃcking of cholesterol and accumulation of unesteriﬁed cholesterol and sphingolipids, such as sphingomyelin and sphingosine, in late endosomes/lysosomes (Vanier and Millat 2003; Lee et al. 2014). Patients with this fatal disease initially develop an ataxic gait and motor dysfunction, typically preceded by vertical gaze palsy and organomegaly, and later develop seizures and dementia. Neurodegeneration, characterized by patterned loss of cerebellar Purkinje cells (PNs) and neuroinflammation (Sarna et al. 2003), is also a feature of NP-C.

A number of experimental disease-speciﬁc therapies, based on the molecular pathophysiology of NP-C, have been tested in cell culture and genetic animal models. Several groups have demonstrated the ability of either centrally or systemically administered therapies alone to reduce the pathophysiology of NP-C (Mellon et al. 2008; Liu et al. 2009; Lee et al. 2010; Wraith et al. 2010). For example, our group demonstrated improvements using histology, with corresponding improvements in neurological function and life span, following bone marrow-stem cell transplantation into the brains of NP-C mice (Lee et al. 2010). Systemically administered therapies have also been found to reduce lysosomal storage in visceral organs, and delay disease progression (Mellon et al. 2008). Long-term treatment with Miglustat has been shown to stabilize neurologic disease, and is well tolerated in adult and juvenile patients with NP-C (Wraith et al. 2010). 2-Hydroxypropyl-β-cyclodextrin (CD) is being studied as an emerging therapy. It chelates cholesterol and has therefore been proposed as a potential therapy for NP-C (Liu et al. 2009); however, it does not cross the blood–brain barrier (BBB) (Pontikis et al. 2013). Therefore, systemic delivery primarily benefits the liver and other organs of the body cavity, whereas therapies that act on the pathophysiology in the brain are needed in order to see substantial neurological improvements. Recently, we have shown that the activity of vascular endothelial growth factor (VEGF) is reduced in NP-C cells, including neurons, and this causes the accumulation of sphingosine that results in the loss of PNs. Increase of VEGF levels in the brain improved the pathophysiology of NP-C by increasing the survival of PNs, motor function, and lifespan, even though the primary genetic defect is not corrected (Lee et al. 2014).

Based on these concepts and ﬁndings, we tested the effects of using a combination of central and peripheral therapies, that is, overexpressing brain-specific VEGF and systemically injecting CD. Here we show for the first time that the effects of combination therapy using VEGF and CD are superior to either central or systemically administered therapies alone on all disease aspects, such as lifespan, PN survival, motor function, lipid correction, and neuroinflammation. We suggest that this combination therapy may provide important new opportunities for the treatment of NP-C.

## Materials and methods

### Animals

**Figure 1.**
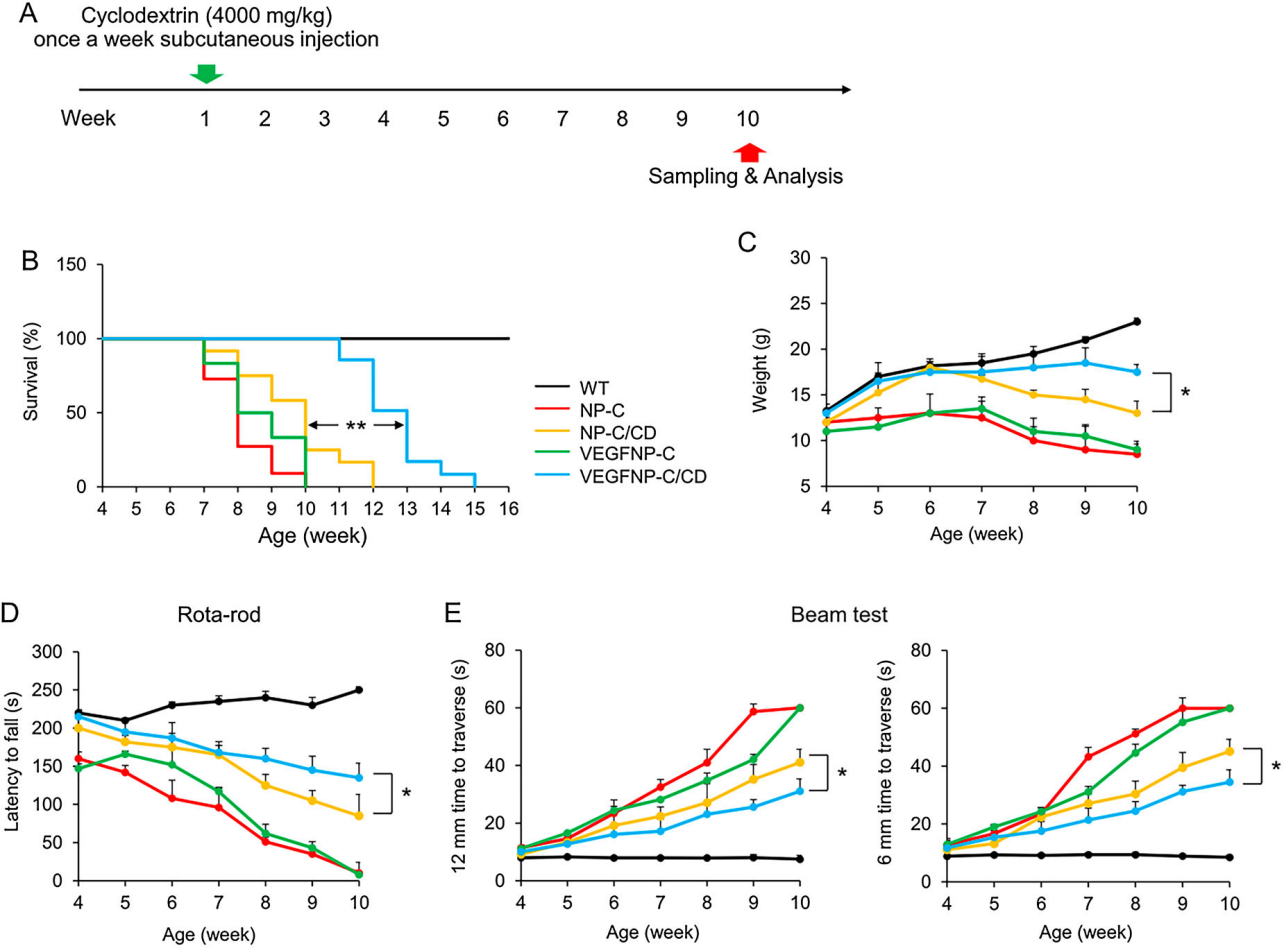
Effect of combination therapy on survival, body weight, and motor function in NP-C mice. (A) Experimental design to determine the effect of combination therapy on NP-C. (B) Survival curve of WT and NP-C mice in each treatment group (*n* = 10–12 per group). (C) Average body-weights of WT and NP-C mice in each treatment group according to age (*n* = 10–12 per group). (D) Rotarod scores of WT and NP-C mice in each treatment group (*n* = 10–12 per group). (E) Beam test of WT and NP-C mice in each treatment group. Left, 12-mm square beam. Right, 6-mm square beam (*n* = 10–12 per group). One-way ANOVA followed by Tukey’s post hoc test. *P < 0.05, **P < 0.01. All error bars indicate s.e.m.

A colony of BALB/c Npc1^nih^ mice was established for this study by brother-sister mating of heterozygous animals. Polymerase chain reaction was performed to determine the genotype of each mouse (Lee et al. 2014). Transgenic mice overexpressing VEGF (Wang et al. 2005) in the brain, under the control of neuron-specific promoters, were bred with NP–C mice to generate VEGF/NP–C (VEGFtg/Npc1^-/-^) mice. Starting at P7 and weekly thereafter, mice were given a subcutaneous injection of cyclodextrin (CD, 4000 mg/kg; H107, Sigma Aldrich, St. Louis, MO). The timeline of the experiment is outlined in Figure 1A. We chose a block randomization method to allocate the animals to experimental groups. To eliminate bias, we were blinded at experimental points such as data collection and data analysis. Mice were housed on a 12 h light–dark cycle with free access to tap water and food pellets. Mouse studies were approved by the Kyungpook National University Institutional Animal Care and Use Committee.

### Behavioral tests

Behavioral studies were performed to assess the balance and coordination of mice by measuring the amount of time the animal was able to remain on a longitudinally rotating rod. Brieﬂy, the Rotarod apparatus (accelerating model 47600; Ugo Basile, Comerio, Italy) was set to an initial speed of 4 rpm, and the acceleration was increased by 32 rpm every 25–30 s. The latency to fall was measured in each trial up to 5 min. Scores were registered every seven days, and three independent tests were performed for each measurement. Motor coordination, balance, and hindlimb placement were evaluated by assessing the ability of mice to traverse two types of balance beams to reach a safety platform. Each mouse was tested for its ability to traverse two different styles of 41 cm long scored Plexiglas beams. One was 12 mm in diameter, and the other was 6 mm wide. Beams were placed horizontally 50 cm above a table. The time taken to traverse each beam was recorded for each trial with a 60 s maximum cutoff, and falls were scored as 60 s. Scores were registered every seven days.

### Immunoﬂuorescence staining

For immunoﬂuorescence staining, brain sections were blocked with phosphate buffered saline (PBS) containing 5% normal goat serum (Vector Laboratories), 2% bovine serum albumin (BSA; Gibco) and 0.4% Triton X-100 (Sigma-Aldrich). Sections were then incubated for 24 h with primary antibodies in the same buffer solution. The following primary antibodies were used: anti-calbindin (rabbit, diluted 1:500; Chemicon, ab82812) and anti-glial fibrillary acidic protein (GFAP, rabbit, diluted 1:500; DAKO). For visualization, sections were incubated with secondary antibodies for 2 h at room temperature followed by PBS washing. Alexa Fluor 488-conjugated goat anti-rabbit IgG (diluted 1:1,000; Molecular Probes, Carlsbad, CA) was used as a secondary antibody. The sections were analyzed with a laser scanning confocal microscope equipped with Fluoview SV1000 imaging software (Olympus FV1000) or with an Olympus BX51 microscope. Metamorph software (Molecular Devices) was used to calculate the average intensities.

### Lipid extraction and sphingosine/sphingomyelin quantiﬁcation

Samples were lysed in a homogenization buffer containing 50 mM HEPES (Gibco), 150 mM NaCl (Sigma-Aldrich), 0.2% Igepal (Sigma-Aldrich) and protease inhibitors (Calbiochem). To quantify sphingosine and sphingomyelin levels, the dried lipid extract was resuspended in 0.2% Igepal CA-630. Four microliters of the lipid extract was added to 20 μl of a naphthalene-2, 3-dicarboxyaldehyde (NDA) derivatization reaction mixture (25 mM borate buffer, pH 9.0, containing 2.5 mM each of NDA and NaCN). The reaction mixture was diluted with ethanol in a 1:3 ratio, incubated at 50°C for 10 min, and centrifuged (13,000 *g* for 5 min). An aliquot (30 μl) of the supernatant was then transferred to a sampling glass vial and 5 μl was applied to an ultra performance liquid chromatography (UPLC) system for analysis. The ﬂuorescence was measured using a model 474 scanning ﬂuorescence detector (Waters). Quantiﬁcation of sphingosine and sphingomyelin peaks was carried out by calculation against the sphingosine and sphingomyelin standard calibration curves using Waters Millennium software.

### Amplex red assay

The cerebellum, liver, lung, kidney, and spleen were lysed with lysis buffer (50 mM phosphate buffer, 500 mM NaCl, 25 mM cholic acid and 0.5% Triton X-100). Unesteriﬁed cholesterol was measured using the Amplex Red Cholesterol Assay Kit (Molecular Probes) according to the manufacturer’s instructions. After incubation for 30 min at 37°C, ﬂuorescence intensities were measured on a microplate reader (Molecular devices) equipped with a ﬁlter set for excitation and emission at 560 ± 10 nm and 590 ± 10 nm, respectively. The cholesterol content of the samples was calculated by measurement against a cholesterol standard curve. Cellular cholesterol content was normalized to protein content.

### Filipin staining

Sections of the cerebellum, liver, lung, kidney, and spleen were ﬁxed with 4% paraformaldehyde for 15 min, washed with PBS and incubated for 30 min with 100 μg ml^−1^ ﬁlipin (Polysciences) in PBS. The sections were washed twice with PBS for 5 min. Images of filipin labeling were obtained using a laser scanning confocal microscope and an Olympus BX51 microscope. Metamorph software was used to calculate the average intensities.

### Statistical analysis

Results are expressed as mean ± standard error of the mean (s.e.m.). Differences between group means were tested for significance using a one-way analysis of variance (ANOVA), followed by a Tukey’s honestly signiﬁcant difference (HSD) post hoc test. Statistical differences between survival curves of groups were determined using a log-rank test. All statistical analyses were performed using SPSS statistical software (SPSS, version 17.0). Differences with a *P* < 0.05 were considered to be statistically signiﬁcant.

## Results

### Combination therapy using brain-specific VEGF overexpression and systemic administration of CD improves survival and motor function in NP-C mice

To examine the therapeutic effects of VEGF, CD and a combination of these, we compared wild type (WT), NP-C, VEGFtg (Wang et al. 2005)/NP-C (VEGFNP-C), CD-treated NP-C (NP-C/CD) and CD-injected VEGF/NP-C mice (VEGF/NP-C/CD) (Figure 1A). Untreated NP-C mice have an acute clinical course and die by 8–9 weeks of age, with a mean survival time of 60 days. Mean lifespan was slightly extended by VEGF overexpression or CD injection alone (mean survival time: VEGF/NP-C = 66 days; NP-C/CD = 68 days), representing an increased lifespan of approximately one week in each case. Combination therapy extended the lifespan more than either therapy alone, and showed an additive benefit, with a 48.5% increase in lifespan above that of the untreated NP-C group (mean survival time: VEGF/NP-C/CD = 89 days; Figure 1B). Another indicator of therapeutic effect in NP-C mice is the maintenance of body-weight. Typically, the body-weights of NP-C mice plateau at 6–7 weeks of age, and then progressively decline. Untreated NP-C, VEGF/NP-C, and NP-C/CD mice showed a precipitous weight loss beginning at 7–8 weeks of age, while CD-treated VEGF/NP-C mice gradually lost weight starting at 9 weeks of age (Figure 1C).

Next, we assessed the motor function/coordination of mice using the Rotarod test. NP-C mice showed a significant decrease in motor function/coordination with age. This was improved by treatment with CD, but was not improved in VEGF/NP-C mice. When compared with other groups, CD-treated VEGF/NP-C mice showed a loss of motor function/coordination that occurred significantly later than in other groups (Figure 1D). Limb coordination and balance were also measured using the balance beam test. NP-C mice took longer to traverse the beams compared with age-matched WT mice. Similar to the Rotarod results, NP-C/CD mice showed an improvement in limb coordination and balance, but this effect was not seen in VEGF/NP-C mice. An enhancement of this effect was observed in CD-treated VEGF/NP-C mice (Figure 1E), indicating the therapeutic synergy of combination therapy.

### Combination therapy delays PN loss and ameliorates neuroinflammation in NP-C mice

**Figure 2.**
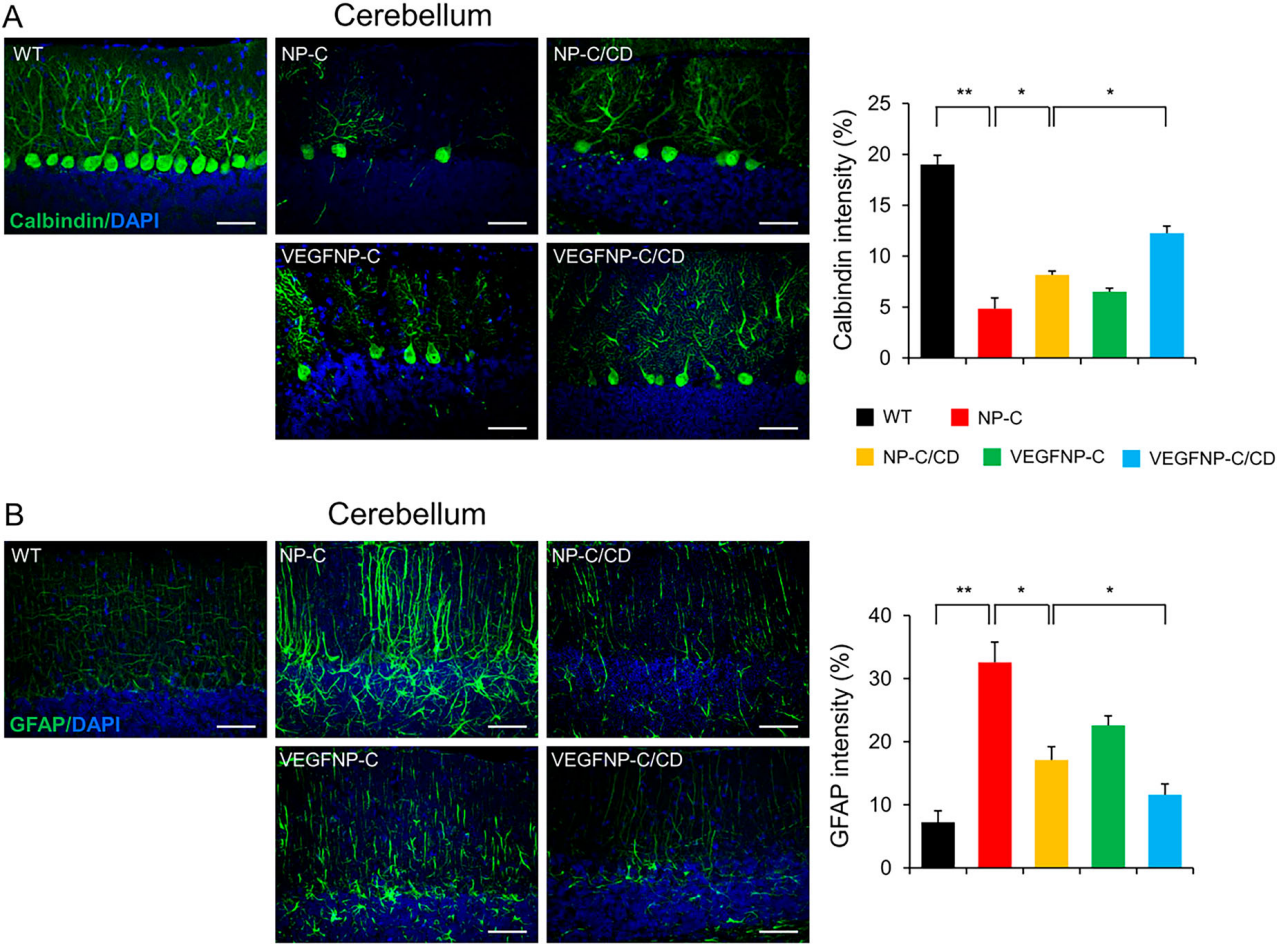
Effect of combination therapy on PN survival and neuroinflammation. (A) Confocal images and quantification of cerebellar PNs in the cerebellum of mice from each treatment group (*n* = 3 per group). Scale bar: 50 μm. (B) Representative fluorescence images and quantification of GFAP in the cerebellum of mice from each treatment group (*n* = 3 per group). Scale bars: 50 μm. One-way ANOVA followed by Tukey’s post hoc test. *P < 0.05, **P < 0.01. All error bars indicate s.e.m.

Since the degeneration of PNs is generally seen in NP-C brain pathology, we quantified PN survival in each of the therapeutic groups. There was limited survival of PNs in the cerebellum of untreated NP-C mice. An increase in the number of PN cells was observed in VEGF/NP-C and NP-C/CD mice (Figure 2A). Remarkably, combination therapy resulted in a high level of neuroprotection when compared with the other groups (Figure 2A). Another feature of brain pathology in NP-C mice is excessive inflammation. To investigate whether combination therapy affects neuroinflammation, we examined astrocytic activation in NP-C mice using GFAP staining. Astrocytic activation was significantly higher in the brains of NP-C mice compared to WT mice, but was decreased in the brains of VEGF/NP-C, NP-C/CD and CD-treated VEGF/NP-C mice (Figure 2B). The greatest reduction in astrocytic activation was observed in CD-treated VEGF/NP-C mice (Figure 2B). Taken together, our results indicate that combination therapy has synergistic effects on neuroprotection in NP-C mice.

### Combination therapy reduces abnormal sphingolipid and cholesterol accumulation in NP-C mice

**Figure 3.**
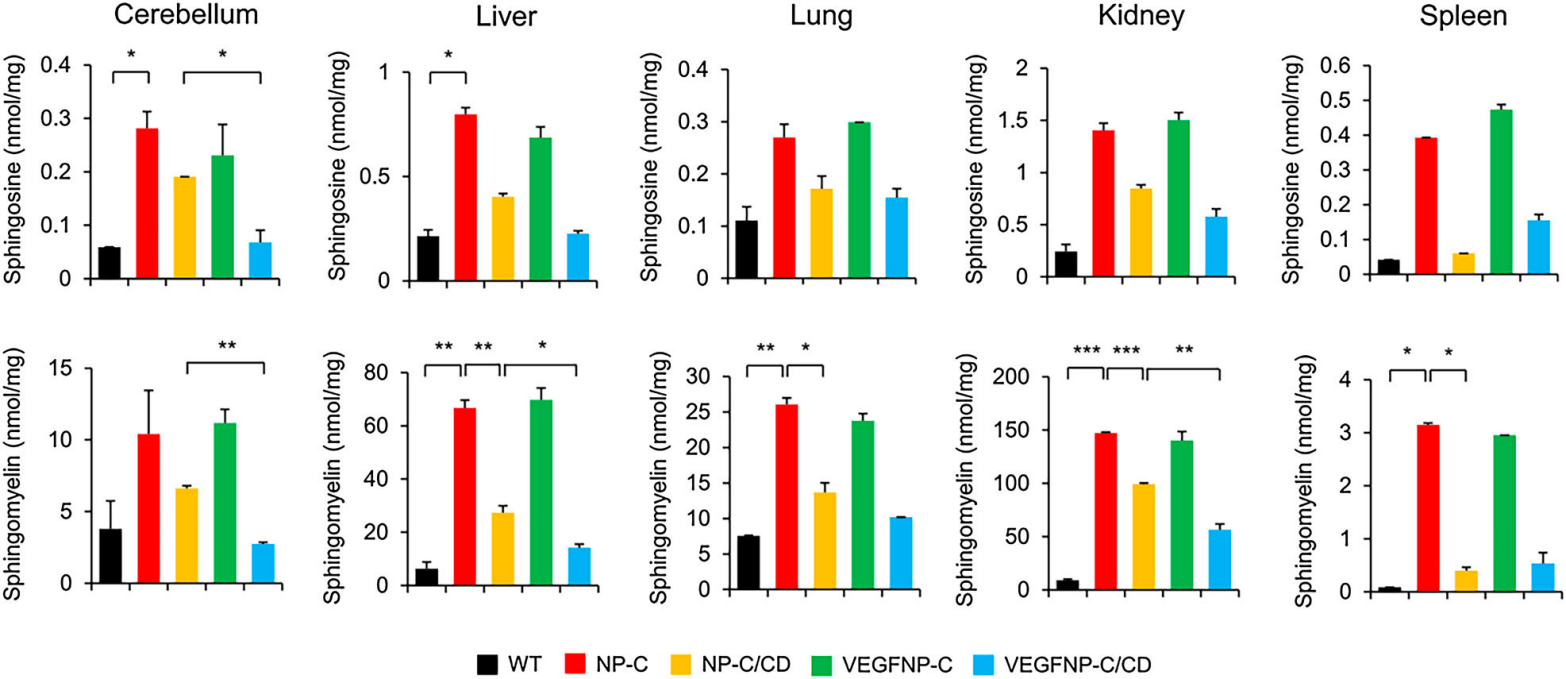
Effect of combination therapy on sphingosine and sphingomyelin accumulation. Sphingosine and sphingomyelin levels in cerebellum, liver, lung, kidney and spleen of each treatment group (*n* = 3 per group). One-way ANOVA followed by Tukey’s post hoc test. *P < 0.05, **P < 0.01, ***P < 0.005. All error bars indicate s.e.m.

**Figure 4.**
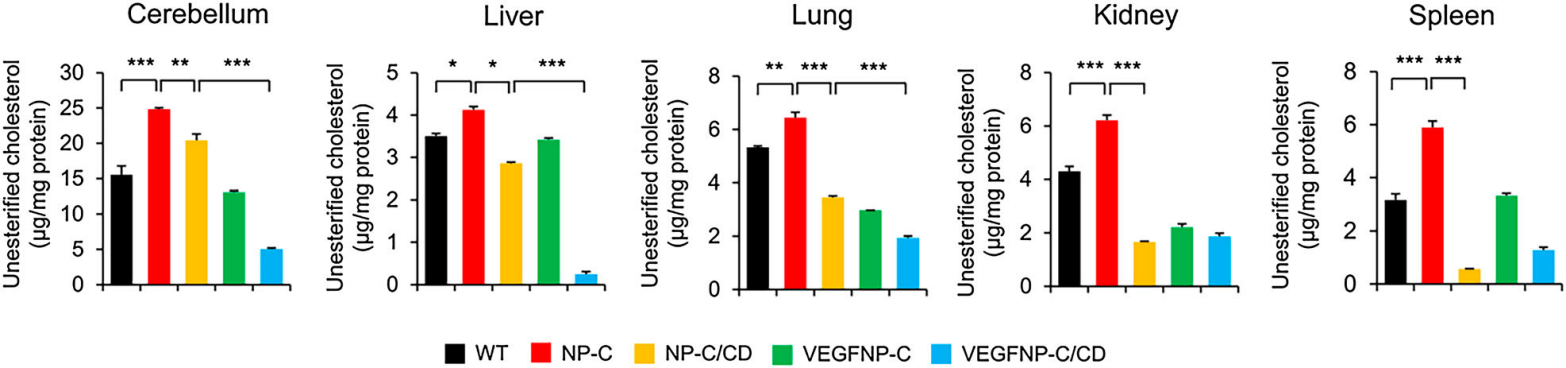
Effect of combination therapy on cholesterol accumulation using Amplex red assay. Unesterified cholesterol levels in cerebellum, liver, lung, kidney and spleen of each treatment group (*n* = 4 per group). One-way ANOVA followed by Tukey’s post hoc test. *P < 0.05, **P < 0.01, ***P < 0.005. All error bars indicate s.e.m.

**Figure 5.**
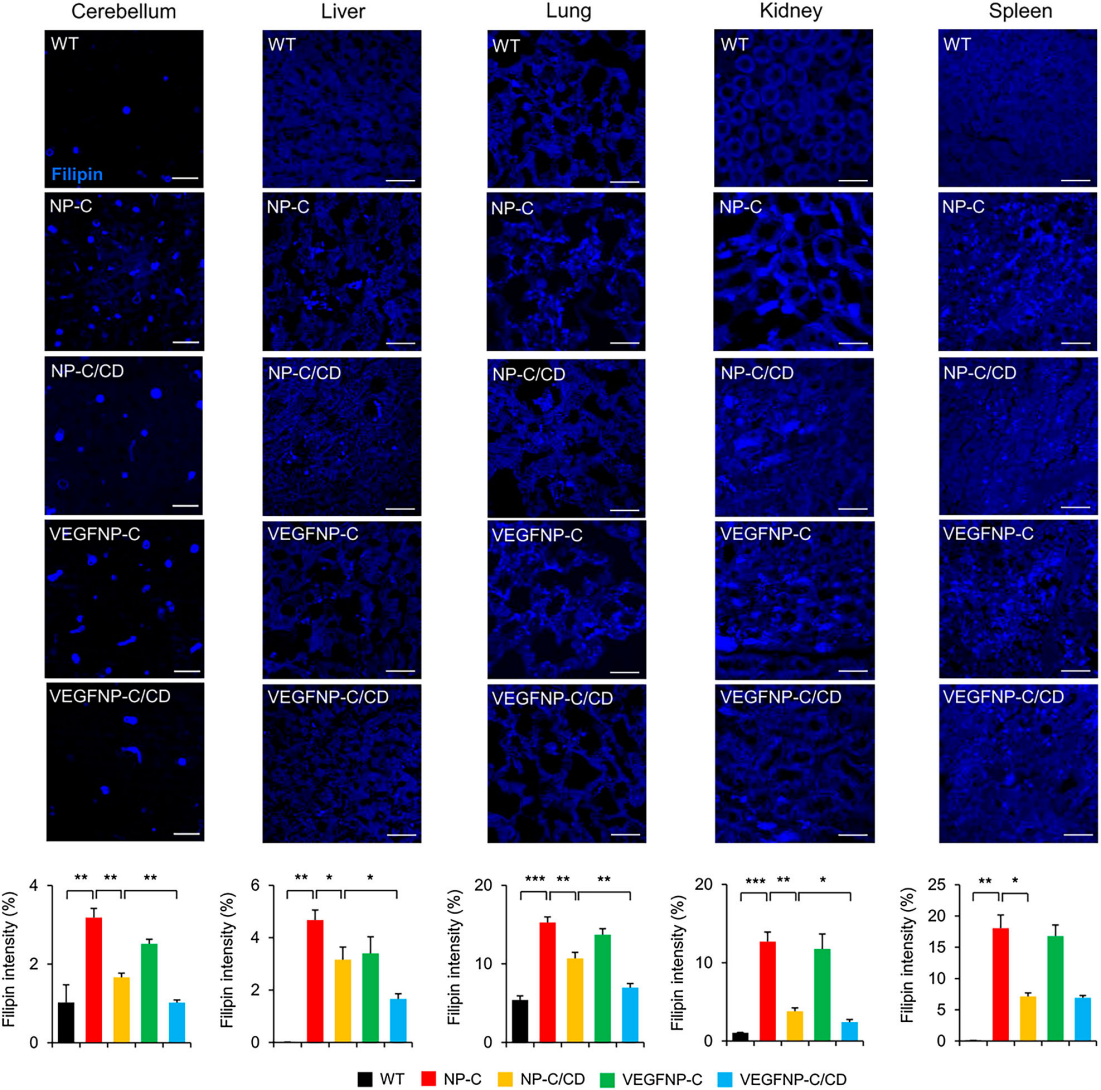
Effect of combination therapy on cholesterol accumulation using filipin staining. Representative images and quantification of unesteriﬁed cholesterol using filipin staining in mice (*n* = 3 per group). One-way ANOVA followed by Tukey’s post hoc test. *P < 0.05, **P < 0.01, ***P < 0.005. Scale bars (cerebellum): 100 μm. Scale bars (liver, lung, kidney and spleen): 50 μm. All error bars indicate s.e.m.

To examine whether genetically increasing brain VEGF expression and systemic treatment with CD affects sphingolipid levels in NP-C mice, we analyzed samples derived from the cerebellum, liver, lung, kidney, and spleen of mice. Compared with WT mice, NP-C mice had significantly increased levels of sphingosine and sphingomyelin in all organs (Figure 3). There was complete improvement of sphingolipid storage in all of the visceral tissues examined from NP-C/CD and CD-treated VEGF/NP-C mice. In contrast, animals overexpressing VEGF in the brain showed a slight decrease in sphingolipid levels when compared with NP-C mice (Figure 3). The sphingolipid levels in the combination therapy group showed a global reduction to WT levels (Figure 3), indicating that VEGF enhances the decrease in sphingolipids induced by systemic administration of CD. Similar to sphingosine and sphingomyelin, unesteriﬁed cholesterol levels were also signiﬁcantly increased in NP-C mice. However, unesterified cholesterol levels were found to be decreased in NP-C/CD, VEGF/NP-C and CD-treated VEGF/NP-C mice, and the magnitude of the reduction was greater in CD-treated VEGF/NP-C mice than other groups (Figure 4), showing a beneficial effect of combination therapy. Decreases in cholesterol levels following the overexpression of VEGF in the brain, systemic administration of CD or combination therapy were further confirmed by filipin staining (Figure 5). Together, our data indicate that combination therapy has a synergistic effect on reducing sphingolipid and cholesterol accumulation in NP-C mice.

## Discussion

NP-C is a highly complex lipid storage disorder that leads to progressive deterioration of the nervous system and multiple organ systems in the body (Vanier 2010; Ottinger et al. 2014). The symptoms of NP-C that present in early childhood include ataxia, seizures, progressive loss of motor function followed by reduced weight gain, cognitive decline, and premature death (Ottinger et al. 2014). The main pathological feature observed in patients with NP-C is the accumulation of cholesterol and sphingolipid in their visceral organs, and also in the brain (Lloyd-Evans et al. 2008). Thus, NP-C has both central nervous system (CNS) and visceral pathologies. Current therapeutic designs use either CNS therapy for the brain or systemic treatments for visceral organs. The therapeutic approach of using CD has been shown to dramatically ameliorate disease symptoms in NPC1 mutant mice (Davidson et al. 2009; Lopez et al. 2014). Intraperitoneal injection of CD decreases cholesterol storage in the liver and causes a slight delay in neurological symptoms in NP-C mice; however, the BBB has been shown to be practically impermeable to CD (Camargo et al. 2001). In addition, systemic administration of Miglustat, allopregnanolone, ibuprofen, and curcumin have shown positive effects in improving the pathophysiology of NP-C (Hovakimyan et al. 2013; Maass et al. 2015). Recently, we have shown that intracerebellar injections of bone marrow stem cells or brain-specific VEGF overexpression can improve the pathophysiology of NP-C (Lee et al. 2010; Lee et al. 2014). However, the exclusive use of either therapy alone may not be optimal in clinical settings because both compartments may need correction to achieve a meaningful quality of life and an increase in the lifespan of patients. Therefore, the present study was designed to determine whether the combination of CNS therapy, using brain-specific VEGF overexpression, and systemic administration of CD provides greater functional benefits and/or enhances neuroprotection in NP-C.

Our findings with regard to treatment with CD are in broad agreement with those of previous studies (Davidson et al. 2009; Pontikis et al. 2013; Ottinger et al. 2014). However, we found that a combination of VEGF overexpression in the brain and systemic administration of CD exhibited a significant synergistic effect in reducing the pathophysiology of NP-C. The benefits of combination therapy were consistently more robust compared to those of VEGF or CD monotherapy alone in all measures. Indeed, combination therapy delayed the onset of ataxic gait, motor dysfunction and weight loss, significantly increased lifespan, and greatly reduced the accumulation of cholesterol, sphingosine, and sphingomyelin when compared with no or monotherapy.

The additive/synergistic benefits gained by combining therapies are also evident in the effects on pathology in the cerebellum. The best-described morphological and characteristic neuropathological feature in NP-C is the dramatic age-dependent loss of cerebellar PNs (Sarna et al. 2003; Vanier and Millat 2003; Lee et al. 2010). It has previously been documented that CD treatment can protect against PN loss in NP-C (Hovakimyan et al. 2013; Maass et al. 2015). The replenishment of VEGF in the brain has also been shown to increase the number of PNs in NP-C (Lee et al. 2014). In line with previous studies, we have observed a protective effect of monotherapy, either using overexpression of brain-specific VEGF or CD injection, in NP-C PNs. Strikingly, there was a strong synergistic increase in PN survival when a dual treatment of CD injection and neuronal VEGF overexpression was used. Neuroinflammation is a common feature found in many disorders, particularly those affecting the CNS. Pathological changes in glial cells have also been reported in mouse models of NP-C (Hovakimyan et al. 2013). Glial cells can induce cytotoxic effects, and glial activation has been considered to be a key process leading to neuronal degeneration in lysosomal storage disorders, such as NP-C (Vanier 2010; Williams et al. 2014). In this study, we observed a decrease in neuroinflammation in the cerebellum of NP-C mice following the overexpression of VEGF in the brain, systemic CD injection, or combination therapy, but the magnitude of the reduction was greater in CD-injected VEGF/NP-C mice (combination therapy) than other groups.

At a cellular level, the mutation in the *NPC1* gene profoundly affects the intercellular trafficking of cholesterol and as a consequence, leads to lysosomal accumulation of multiple lipid species (Vanier and Millat 2003). This increased lipid storage is observed in almost all tissues, characterized by marked elevations in unesterified cholesterol and complex sphingolipids, including sphingomyelin and sphingosine (Vanier and Millat 2003; Fan et al. 2013). The complex lipid storage events in NP-C disease, like other sphingolipidoses, are also associated with alterations in neuronal function (Walkley and Suzuki 2004), and deterioration of diverse organ systems in the body (Vanier 2010). We found that the abnormalities in sphingolipid and cholesterol accumulation were decreased by systemic treatment with CD or the overexpression of VEGF in the brain, although moderate changes were observed with VEGF replenishment. Compared with monotherapy, combination therapy induced a significant decrease in sphingolipid and cholesterol accumulation, indicating that dual therapy has synergistic effects on restoring lipid levels to a normal range.

While targeting the initial gene/protein defect in NPC1 may prove elusive for the time being, the presence of many different downstream therapeutic targets would suggest that a combination of central and peripheral therapies might play an important role in the management of symptoms for patients with NP-C, and could potentially have synergistic effects. Here we show for the first time that combining the overexpression of brain VEGF with the systemic administration of CD to treat the pathogenic cascade has enhanced therapeutic beneﬁts in a mouse model of NP-C. While additional studies are required to identify the exact mechanisms at play, the outcome of using VEGF/CD combination therapy is clearly more beneficial than using a single treatment strategy. Combination therapy leads to a delay in the onset of clinical signs, a significant increase in lifespan, a reduction in cholesterol and sphingolipid accumulation, diminished neurodegeneration, and normalization of neuroinflammatory mediators. Taken together, our results demonstrate the amenability of NP-C to combination therapy, and suggest new therapeutic options for NP-C patients.
